# The future of human malnutrition: rebalancing agency for better nutritional health

**DOI:** 10.1186/s12992-021-00767-4

**Published:** 2021-10-09

**Authors:** Jonathan C. K. Wells, Akanksha A. Marphatia, Gabriel Amable, Mario Siervo, Henrik Friis, J. Jaime Miranda, Hinke H. Haisma, David Raubenheimer

**Affiliations:** 1 Childhood Nutrition Research Centre, Population Policy and Practice Research and Teaching Programme, UCL Great Ormond Street Institute of Child Health, 30 Guilford Street, London, WC1N 1EH UK; 2 Department of Geography, University of Cambridge, Cambridge, UK; 3 School of Life Sciences, University of Nottingham Medical School, Queen’s Medical Centre, Nottingham, UK; 4 Department of Nutrition, Exercise and Sports, University of Copenhagen, Copenhagen, Denmark; 5 CRONICAS Centre of Excellence in Chronic Diseases, Universidad Peruana Cayetano Heredia, Lima, Peru; 6 Department of Medicine, School of Medicine, Universidad Peruana Cayetano Heredia, Lima, Peru; 7 Population Research Centre, Department of Demography, University of Groningen, Groningen, the Netherlands; 8 Charles Perkins Centre, University of Sydney, Sydney, Australia

**Keywords:** Dual burden of malnutrition, Agency, Undernutrition, Stunting, Obesity, Food systems, Social inequality

## Abstract

The major threat to human societies posed by undernutrition has been recognised for millennia. Despite substantial economic development and scientific innovation, however, progress in addressing this global challenge has been inadequate. Paradoxically, the last half-century also saw the rapid emergence of obesity, first in high-income countries but now also in low- and middle-income countries. Traditionally, these problems were approached separately, but there is increasing recognition that they have common drivers and need integrated responses. The new nutrition reality comprises a global ‘double burden’ of malnutrition, where the challenges of food insecurity, nutritional deficiencies and undernutrition coexist and interact with obesity, sedentary behaviour, unhealthy diets and environments that foster unhealthy behaviour. Beyond immediate efforts to prevent and treat malnutrition, what must change in order to reduce the future burden? Here, we present a conceptual framework that focuses on the deeper structural drivers of malnutrition embedded in society, and their interaction with biological mechanisms of appetite regulation and physiological homeostasis. Building on a review of malnutrition in past societies, our framework brings to the fore the power dynamics that characterise contemporary human food systems at many levels. We focus on the concept of agency, the ability of individuals or organisations to pursue their goals. In globalized food systems, the agency of individuals is directly confronted by the agency of several other types of actor, including corporations, governments and supranational institutions. The intakes of energy and nutrients by individuals are powerfully shaped by this ‘competition of agency’, and we therefore argue that the greatest opportunities to reduce malnutrition lie in rebalancing agency across the competing actors. The effect of the COVID-19 pandemic on food systems and individuals illustrates our conceptual framework. Efforts to improve agency must both drive and respond to complementary efforts to promote and maintain equitable societies and planetary health.

## Introduction

Until around 12,000 years ago, all human populations foraged for diets comprising wild foods. Nomadic foraging represented a broadly common social system, though subsistence practices varied by ecology and geography; and aside from the systematic use of tools and fire the basis of nutrition was not markedly different from that of other social primates. Since the beginning of the Holocene, however, human populations have to various extents undergone several cumulative revolutions, first in the emergence of different types of agriculture, then urbanization followed by industrialization and technological innovation, and finally globalization and the digitalization of many aspects of life. Throughout these revolutions, through which the overall human nutritional niche has been steadily reconstructed, the persistence and unequal distribution of malnutrition has remained a strong signal [1].

Scientific efforts to treat or prevent malnutrition have themselves evolved with the social priorities and dominant health challenges of the day. Early efforts targeted undernutrition, closely associated with poverty, infections and restricted diets. Today, however, the dominant manifestation comprises obesity, though undernutrition persists globally. The co-existence of these conditions, first observed at the population level, has been termed the ‘double burden of malnutrition’ (DBM) [2]. Recently, it has become apparent that many individuals also experience both nutritional extremes at different periods of the life-course, or even simultaneously as in the case of obesity and micronutrient deficiencies [3]. Ostensibly, the risk factors for undernutrition and obesity seem very different, but there are many common drivers [1, 3–5].

Importantly, malnutrition in all its forms is increasingly linked with other major challenges facing our species. For example, at the population level there are common drivers of undernutrition, obesity and climate breakdown [5], hence human malnutrition is fundamentally linked with planetary dysfunction. A key issue, currently attracting substantial attention, is how we feed a projected global population of ~ 10 billion by 2050 in ways adequate for the health of both people and planet [6]. The DBM is also closely linked with many aspects of ongoing globalization and associated nutrition transition [3, 4], which are likewise implicated in climate breakdown [5].

In the short-term, many different efforts have aimed to treat or prevent different forms of malnutrition, either through targeting malnourished individuals directly, or through preventive public health efforts that typically attempt to promote healthy diets and exercise while reducing environmental stresses such as infections. Here, we take a longer-term view, and consider what must be achieved if we are to see a substantial reduction in the global burden of malnutrition in all its forms in the future.

To develop this perspective, we articulate a conceptual framework that focuses on the deeper structural drivers of human malnutrition embedded in society. Whatever the contribution of ecological volatility, it has been recognized since Sen’s work in the 1980s that famines primarily represent the failure of societies to distribute food equitably [7]. We now need to reconsider Sen’s insight in the context of the DBM and globalized food systems. To promote healthy people, we need healthy societies, recognizing the primary role played by food systems in the construction and the functioning of all human communities [1]. This turns attention on the way that socio-economic systems and food systems are mutually embedded, with profound consequences for all aspects of food production, distribution and consumption. Although broader facets of the food system are widely understood to impact nutritional status and behavior at the individual level [1, 2, 4, 5], research on the underlying physiological and behavioral mechanisms would benefit from better integration with our understanding of societal dynamics.

Our review therefore has five main aims. First, we set out a broader conceptual model of nutrition, that can provide a robust framework with which to imagine a better future. Second, we use this framework to critically examine how we got to where we are today, by looking at the long-term history of malnutrition. Third, we summarize the current manifestation of malnutrition and its associations with fundamental societal drivers. Fourth, we highlight the complex role of agency in malnutrition, focusing on how our biological drives are impacted by a ‘competition of agency’ between multiple actors. Using this approach, we highlight nutrition as a key pathway through which structural factors ‘get under the skin’ and damage health. We illustrate this framework by focusing on the COVID-19 pandemic. Finally, based on these insights, we review future opportunities to prevent and treat malnutrition.

## A broad definition of nutrition

**Fig. 1:**
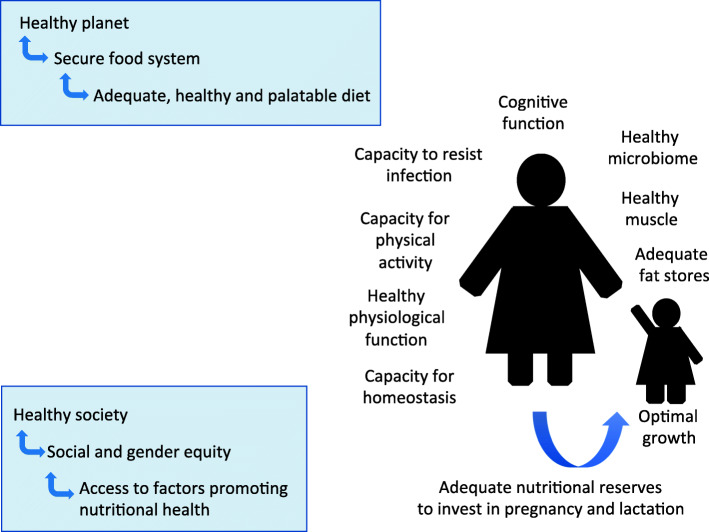
A broad conceptual model of the remit of human nutrition. In addition to dietary intake, nutritional health relates to functional capacities of the body, and a level of physical activity that maintains healthy metabolism. Healthy nutrition in one generation is essential for a healthy pattern of development in the next generation. Nutritional health at the individual level then depends on interacting with healthy societies that are compatible with planetary health

To underpin this discussion, our approach requires a broad definition of nutrition (Fig. 1).

We need to go beyond the conventional remit of ‘what we eat’, to consider more broadly aspects of inequality in ‘how we are nourished’. This perspective allows us to consider what is needed from a society that would enable individuals to be free from all forms of malnutrition.

According to the Sustainable Development Goals, healthy societies may be considered to achieve each of ‘ecological health’, ‘wellbeing’, ‘social equity’, and ‘economic prosperity’ [5]. Nutrition is understood to be integral to each of these outcomes [1, 5, 8–13], but in this context means and ends are often confused [14], and the current role of nutrition in promoting economic prosperity works directly against its role in the other three dimensions.

At the level of the individual, we propose that nutritional health involves not only adequate quantity and quality of food intake, but also healthy physical activity levels, optimal growth from conception to adolescence, healthy body composition, the ability to maintain homeostasis and resist infections, and the capacity for women to adequately nourish the next generation during pregnancy and lactation, and thereafter.

Considering only this individual level, it is immediately clear that large numbers are unable to meet such a definition of health. In 2018, among children under 5 years of age, 150 million globally were stunted (low height for age), 50 million wasted (low weight for height), and 38 million had obesity, while over 2.1 billion adults had overweight or obesity [15]. From an evolutionary perspective, the human nutritional niche is impacting our survival, health and longevity, while also driving major inequalities in these outcomes.

Beyond the individual level, it is increasingly understood that malnutrition is embedded in unhealthy economies and societies, as well as planetary dysfunction [1, 5]. To address this burden, we need to reframe the problem within an integrated scientific understanding of the full range of causal factors, and identify the subset that is most amenable to managing for change. We argue that the issue of ‘agency’ transcends all of these causes and opportunities.

Whatever form society takes, nutrition depends fundamentally on ‘agency’. At the level of the individual, we define agency as *the capability of individuals to pursue their goals* [16, 17]. It is important to note that the range of ‘goals’ goes far beyond the simple relationship between dietary intake and personal health. Individuals maximize a wide range of goals related to food, including enjoyment, convenience, expression of identity, socializing and financial management. Moreover, choices related to food are often made in the context of ensuring the nutrition of others, such as younger and elderly age groups, or those with various forms of vulnerability and disease.

In the specific context of nutritional health, the expression of agency translates into the capability to obtain adequate quantities of a nutritious diet, while also having the physiological capacity and cognitive skillset to defend against societal and ecological causes of malnutrition. At the population level, collective agency should enable societies to create food environments that are sustainable for human and planetary health, and that protect against malnutrition [17]. As we show later in this article, however, many aspects of human food systems act directly and intentionally to *distort or reduce* agency at the level of individuals and populations, and are embedded in many forms of inequalities [1]. The collective agency of various organizations, including corporations, governments, and supranational institutions must therefore also be taken into account.

Using this broad model, we can revisit what is needed of human societies to reduce all forms of malnutrition. In order to improve understanding of where such efforts should be targeted for greatest efficacy, we first need to improve understanding of why malnutrition has persisted in different forms across time, geography and society. We show in the next section that the history of malnutrition is also fundamentally the history of constraints on individual agency.

## The history of malnutrition

In non-hierarchical societies, nutrition is determined largely through individual agency, expressed as the interaction of appetite, social factors and environmental food availability. In the distant past, for example, Paleolithic foragers living in small social groups were able to achieve relatively large body size and nutritional health through consuming diverse diets of vegetables, tubers, fruits and meat, providing high intakes of protein, fiber and micronutrients [18, 19]. Evidence that Paleolithic populations engaged in feasting reminds us that nutrition has long had a critical social dimension [20, 21].

However, all human communities can potentially express common phenotypes of thinness and overweight, as indeed can many non-human primate species [22]. This indicates that ecological stresses were sufficiently common during primate evolution to have favored mechanisms of metabolic and behavioral plasticity. In any era, malnutrition emerges when adverse environments or food systems constrain these plastic mechanisms.

Beyond natural ecological volatility and the associated risk of food shortages, a new burden of human undernutrition emerged with the origins of agriculture. Over the past 12,000 years or so, the domestication of numerous species of plants and animals occurred independently and in different ways in different parts of the world, though a small proportion of humanity continues to practice hunting and gathering [23]. While the transition to agriculture may have increased the overall supply of food-energy, sedentary farmers were also inherently more susceptible than foragers to periodic undernutrition, being less mobile and dependent on a narrower range of foodstuffs, whilst also exposed to famines and higher pathogen burdens [23]. The skeletal record post-agriculture shows near-universal falls in linear bone lengths and increased markers of bone disease, indicating dietary inadequacies, repetitive physical activities related to growing and processing food, and elevated infectious burdens [23–26].

These stresses appear to have been most challenging when associated with the emergence of early states and hierarchical societies, which regulated access to the land and demanded from individual farmers a proportion of their harvest [27]. While foraging societies tend to constrain social differentiation, by pooling risks within and across social groups [28], farming allows new relations of inequality to emerge. At the level of individual households, early farmers were at risk of harvest failure, and of being unable to meet their obligations. The resulting debts often led to the loss of their land rights and agricultural capital. Over time, this led to the divergence of classes of landowners and disempowered tenant farmers, or peasants [29]. From a broader perspective, the emergence of differentiation in subsistence strategy is not unique to humans: many species display complementary strategies of ‘producing’ food, or ‘scrounging’ it from other producers [30, 31]. Even if a strategy of ‘all producing’ generates the most equitable division and largest supply of food within a population, scrounging is predicted to emerge as soon as any individual producer can increase their returns by switching strategy [32]. For humans, this scenario generates a paradox that when farm productivity rises, egalitarian food production may inherently represent an unstable scenario. Consistent with that hypothesis, different forms of farming gave rise to many forms of social inequality [1].

In particular, early states sought to control large numbers of peasants, and across different global regions achieved this by converging on forms of grain agriculture [27]. Given intensive labor inputs, grains produce high yields and the harvests are easy to store and transport. This made them ideal for state taxation, but at the same time exposed their producers to high physical workloads in combination with diets low in protein and micronutrients, and hence increased the risk of chronic undernutrition [27]. Moreover, by controlling access to the land, elites and states proactively used the threat of hunger to coerce peasants to produce food for both landowners and peasants. To augment both the territory and the workers under their control, states also regularly invaded their neighbors [27], and deliberately used starvation in the form of sieges as a routine military strategy [1]. In these early forms of stratified societies, therefore, farming structurally connected the production of food with the control of large numbers of people through hierarchical relations.

In such societies, the primary defense against malnutrition comprised different ways of preserving or enhancing individual or collective agency. When the level of inequality and hierarchy became intolerable, or during periods of political instability, many farmers fled back to more marginal habitats and grew crops less amenable to taxation [27]. In ancient Greece, however, a different resolution emerged: competition between landowners and tenant farmers spurred the emergence of early democratic institutions, freeing the farmers from their obligations to provide food for the landlords, and recasting them as politically active citizens with new rights and social duties [33]. We highlight this as a way in which early state societies could reorganize themselves along more equitable lines, though it is important to note that these benefits did not reach all individuals, and that Greek society continued to use slave labor.

In ancient Rome, however, democratic institutions did not develop in this way, and the majority of citizens remained susceptible to economic uncertainty, hunger and debt. Roman agriculture remained fundamentally based on slave labor, and the expansion of the empire was explicitly driven by the aim of increasing the number of slaves. Instead of empowering its urban citizens, the Roman state simply provided food handouts during subsistence crises [25]. Roman law, with its emphasis on private property, has subsequently been influential in shaping global institutions, and has played a key role in underpinning restrictions on individual agency as market economies developed [33].

Even in the ancient world, food systems of different global regions were highly connected. Trade in luxuries such as spices was closely associated with trade in other commodities, including slaves [34]. From the medieval era onwards, food systems in different global regions began to become further inter-connected, and underwent a series of changes that cumulatively exacerbated both societal and geographical inequalities. A mercantile system, involving the import of tropical spices into Europe and the export of slaves from Africa to New World plantations, evolved into a system where Europe received large quantities of agricultural commodities produced in different global regions by European settlers, indentured laborers or farmers from colonized countries [35, 36]. At every stage, the production of food continued to involve major constraints on the agency of those producing it.

Similarly, despite increasing food availability in wealthier countries as they began to industrialize, the threat of hunger continued to be used to coerce the new classes of industrial worker [37]. Access to the land was steadily reduced for rural populations, propelling them to rapidly-growing cities where they provided paid labor in the new factories. The provision of low wages by the new industrialists coerced these laborers to work long hours in order to earn enough to cover basic food requirements, and chronic undernutrition was widespread. By the late nineteenth century, it was increasingly recognized that this burden of undernutrition was itself undermining industrial productivity, and new public health efforts were introduced to improve working conditions and diets [38, 39]. These efforts were consolidated in the aftermath of World War II, when it was clear that the entire global food system needed reconfiguration [40].

Despite these efforts, geographical inequalities persisted and took on new forms in the post-war era. The new international order initiated at the 1944 Bretton Woods conference aimed to stabilize the global economy, while ensuring that high-income countries (HICs) had access to the raw materials, markets and consumers that drive their national economies. To operationalize this system, new international financial institutions (IFIs) were created, such as the World Bank, International Monetary Fund (IMF) and World Trade Organization (WTO). These IFIs reduced the ability of governments of the formerly colonized nations to organize food production and consumption in the interests of their newly independent populations, while also empowering new transnational corporations (TNCs) [41]. Throughout these transformations, the agency of groups and organizations representing individual food producers and consumers was persistently subordinated to the interests of larger-scale corporate organizations. Renewed concern over global undernutrition in the 1970s stimulated the Green Revolution, applying new technologies to selected crops. This effort increased farm yields, but maintained structural inequalities [42, 43].

Undernutrition remained the primary human nutritional stress for millennia, but there is also ancient evidence of corpulence. The earliest evidence relates to Venus figurines from the European Paleolithic [44], that provide sufficiently accurate depictions of the human body to indicate direct experience of female overweight in this era. These figurines are widely interpreted as expressing positive attitudes to large body size in women, though the specific reasons remain unclear, and there is no evidence of how this may have related to ill-health. However, by the early historical era, medical authorities in ancient Greece and Rome clearly recognized obesity as an undesirable condition that was detrimental to health, and developed treatments [45]. Overweight is generally considered to have remained relatively rare until recent centuries, and to have been restricted to elites, though relevant evidence remains scarce. Long-term systematic increases in average body mass index (BMI), and in the prevalence of overweight, are evident from the nineteenth century in HICs [46], and have accelerated in every global region during the last half-century [47]. The obesity epidemic initially affected wealthier groups but to varying degrees across countries is now increasing faster among poorer groups [48, 49], reflecting a global shift towards unhealthy diets and sedentary behavior.

This brief history of malnutrition helps contextualize its current global manifestation. Given our unique agricultural niche, human nutrition is inherently sensitive to ecological shocks, but ever since the emergence of state societies the most powerful driver of malnutrition has been societal dynamics. At both local and global levels, the evolution of human food systems has always been fundamentally intertwined with the evolution of hierarchical politico-economic systems. These systems evolved to control populations as well as to feed them [1], and the primary change over time has been in how particular food systems achieve this control. Regarding both food production and consumption, the systematic suppression of individual agency underlies the persistence of malnutrition-inducing/enhancing environments. This relationship remains evident if we consider malnutrition in contemporary populations.

## Contemporary manifestation of malnutrition

**Fig. 2:**
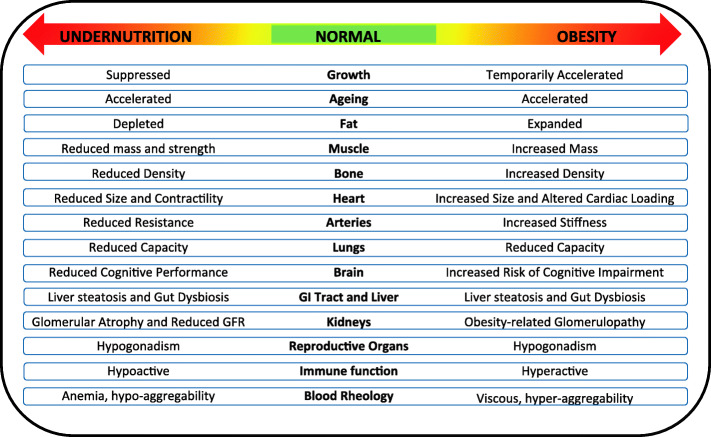
Undernutrition and obesity impact adversely at many biological levels. Both forms of malnutrition affect the morphology and functioning of many individual organs and tissues, as well as growth, ageing rate, and the composition and functioning of the microbiota

Contemporary malnutrition incorporates both deficiencies and excesses in diverse aspects of nutritional status including dietary intake, nutrient status, tissue masses, and physical activity [50–52]. Crucially, both extremes of malnutrition impact adversely across many different levels of biology (Fig. 2). Undernutrition remains a major risk for child mortality [37] and reduces human capital [43], while the DBM is the primary biological driver of the emerging global epidemic of non-communicable diseases (NCDs) [3]. The health penalties are exacerbated when the DBM manifests within individual life-courses, as the toxic effects of obesity on NCD risk are enhanced among those who also experienced undernutrition in early life [3]. Globally, the number of premature deaths per year attributable to dietary risk factors is estimated to be 11 million, and the number of ‘disability-adjusted life-years lost’ to be 255 million [53]. The prevalence of undernutrition is decreasing slowly, though large numbers of children remain affected, while that of overweight and obesity is rising among children and adults in every geographical region [15].

In settings where child undernutrition is common, a key proximate cause relates to monotonous diets based on starch-rich staples, that provide limited intakes of energy, micronutrients and protein. However, the broader environment is also important. Nutrient deficiencies and exposure to pathogens and toxins may in combination impair the absorptive capacity of the gut and cause intestinal and systemic inflammation [54]. Traditionally, conceptualization of the resulting child undernutrition differentiated ‘chronic’ versus ‘acute’ conditions. The latter, indicated by low tissue mass (wasting), implies a need for immediate nutritional rehabilitation, whereas linear growth retardation, eventually manifesting as ‘stunting’, was considered a marker of chronic undernutrition that would not respond to nutritional treatment. However, it is increasingly understood that the two forms are closely related [55], with each of wasting and stunting increasing the risk of the other developing over time [56]. Moreover, a recent study across 84 low- and middle-income countries (LMICs) found that 3% of young children are simultaneously wasted and stunted, resulting in particularly high mortality risk [57]. Precisely because it reflects exposures more distal than immediate food intake, the epidemiology and ontogenetic development of stunting provide unique insight into the broader causes of undernutrition.

Stunting emerges from composite ‘cycles of disadvantage’, bringing together several ecological and societal stresses that are embedded in social inequity and that propagate across generations [1]. These stresses impact nutrition and growth during the first ‘thousand days’ of life, and thereby shape adult size, body composition and health profile, as well as biological traits in the next generation [58–60].

In susceptible populations, growth faltering is typically already evident at birth, indicating undernutrition in utero [61]. From an evolutionary perspective, early growth faltering reflects both inadequate maternal nutrition, but also the diversion of nutritional resources away from growth to other biological functions. In post-natal life, for example, linear growth may be traded off first against immune function [62] and subsequently against earlier reproduction [63]. Those under-nourished in early life are prone to develop central adiposity if they subsequently gain excess weight [64], which may reflect the role of visceral fat in promoting immune function [65, 66]. In a prospective Brazilian birth cohort, for example, a composite marker of low ‘maternal capital’ (incorporating education, height, BMI and family income) was associated with poor linear growth, higher BMI, more central fat distribution and early childbearing in the daughter [67]. These associations remind us that growth variability emerges as part of more comprehensive biological responses to prevailing ecological conditions.

**Fig. 3:**
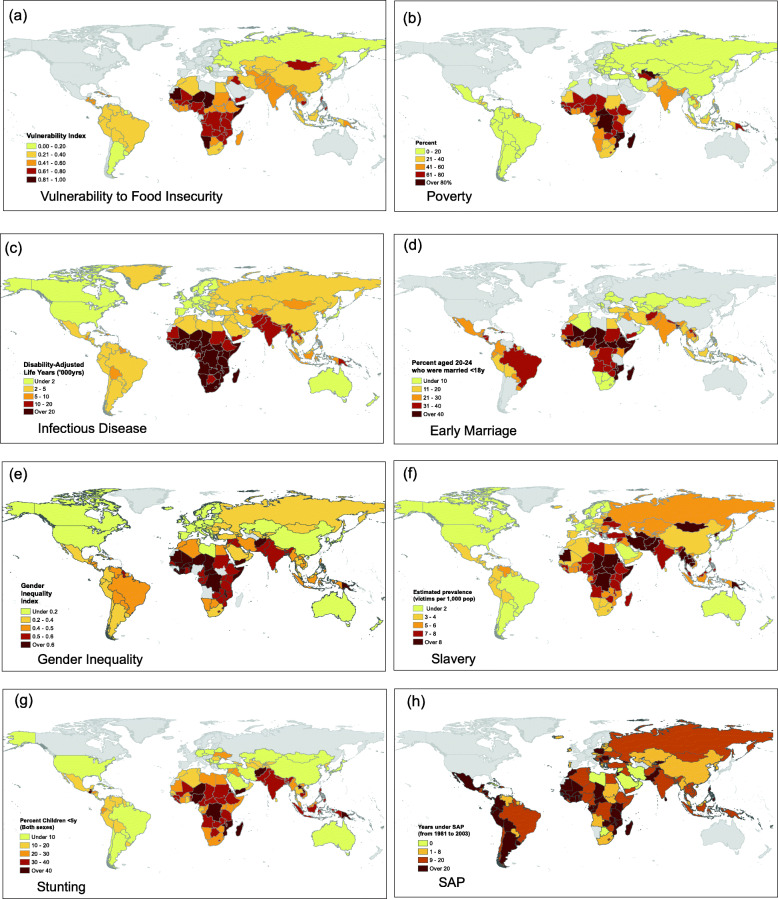
Multiple components of adversity are geographically clustered across low and middle-income countries. Persistent socio-ecological stresses include (**a**) food insecurity and vulnerability to climate change; (**b**) poverty measured as the proportion of the population living on <USD 3.1 per day; (**c**) infectious disease burden assessed as the disability-adjusted life years per 100,00 population attributable to communicable, maternal, neonatal, and nutritional diseases; (**d**) prevalence of marriage < 18 years among women aged 20–24 years; (**e**) women’s disadvantaged status in society, measured by the Gender Inequality Index; and (**f**) coerced labour, assessed as the estimated prevalence of slavery per 1000 population; Maps (**a**) to (**f**) show similarity to (**h**) the prevalence of stunting, a composite marker of undernutrition, categorised as height z-score < − 2. (**g**) The same countries have experienced exposure to economic liberalisation, assessed as the number of years subject to structural adjustment programs between 1981 and 2004. Data from ‘Our World in Data’ or [68–73]

At a global level, the geographical distribution of stunting closely replicates that of many specific markers of disadvantage (Fig. 3). Importantly, most of these markers reflect the dynamics and norms of human societies, all indicating reduced individual agency. However, LMIC populations with high levels of these challenges are now also increasingly exposed to the impacts of globalization and nutrition transition. This means that populations with high levels of undernutrition are now also experiencing an increased availability of cheap highly processed foods, alongside other unhealthy commodities and drivers of sedentary behavior [74].

Through nutrition transition, diets tend to increase in energy, refined carbohydrate and fat content, while lacking adequate protein, fiber or micronutrients [75, 76]. These shifts may simultaneously drive excess energy consumption while maintaining nutrient deficiencies. There is growing evidence, for example, linking diets high in industrially-processed foods both with poor infant and child growth [77–80], and with obesity from childhood onwards [81–83]. Crucially, the global nutrition transition is rapidly outpacing public health success in resolving undernutrition, so that obesity is increasing faster than stunting is decreasing [15].

Exposure to heavily processed industrial foods is closely associated with international trade patterns and the activities of TNCs, which benefit from trade liberalization [84, 85]. For many LMICs, trade liberalization was a key condition of receiving support from IFIs during economic crises [86]. Figure 3 highlights that the global regions prone to cycles of disadvantage are also those with long-term exposure to such conditionalities. This clustering of environmental, social and economic factors contributes to the speed of nutrition transition in many countries, as we discuss in more detail below.

Moreover, recent analyses show that the DBM is emerging at lower levels of economic development, both across and within countries, as processed foods become more widely available and cheaper [2]. This is causing rapid shifts in the population groups most affected by obesity, whereby it first emerges in wealthier group but then becomes most prominent in poorer groups [49, 87]. Secular trends currently manifesting in LMICs are less in height, and more in BMI and, in females, earlier menarche [3, 47, 88]. Initially, obesity rates rose fastest in urban LMIC populations, but recently this shifted to rural populations [89], reflecting the growing penetration of nutrition transition into rural areas [10].

To understand why undernutrition and obesity increasingly co-exist not only within communities and households but within individuals through the life-course, and why obesity is increasing in prevalence faster than undernutrition is decreasing, we next develop our framework to demonstrate how the contemporary nutrition transition is related to the agency of both individuals and various types of organization.

## Agency

Human nutrition is embedded in complex power dynamics operating at many levels of society, involving a ‘competition of agency’ between multiple actors [1, 5, 90]. To fully understand how these power dynamics drive the DBM, we need to consider how this competition of agency interacts with the physiological drives that underpin appetite and eating patterns. In setting out this conceptual model, we want to emphasize that a degree of agency pertains to each type of actor, and that no actor is entirely devoid of agency. At the same time, the notion of competition highlights the fact that the agency of any one type of actor may to varying degrees be constrained or manipulated by the agency of other types. We illustrate these issues in more detail below.

To illustrate these dynamics, we focus here primarily on the role of highly-processed industrial foods. These are not the only relevant dietary factors, but importantly, they have been linked with both extremes of malnutrition. If we conceive of the global food system as a ‘dynamic societal game’ [1] and the nutritional status of individuals as the key biological outcome, then our aim here is to understand the different actors involved, the ‘rules of the game’, and how and to what extent each type of actor can express agency. This will enable us to explore how broader structural factors ‘get under the skin’ to harm health, through the medium of different forms of malnutrition. We start with the component of agency that is embedded in our biology, our appetite systems.

### Biological drives

At the level of physiology, individual agency is regulated through multiple components of homeostasis [91]. Physiological systems can be characterized as goal-directed entities organized to maintain or attain particular states in the face of external variation. Regarding nutrition, the key regulatory systems concern appetite. Across diverse species, including humans, the body satisfies its requirements for protein, fat, and carbohydrate (as well as some micronutrients) via specific appetites that detect deficiencies and surpluses and motivate feeding behavior accordingly [92].

**Fig. 4:**
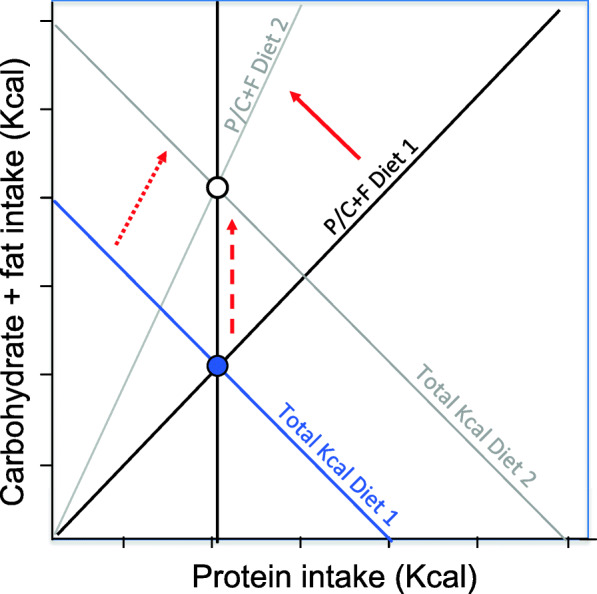
Schematic illustration of the protein leverage effect. The solid blue circle shows the bi-coordinate regulatory target for protein, non-protein energy (carbohydrate and fat) and total energy (the blue negative diagonal) in a hypothetical reference diet (Diet 1). When protein is diluted with carbohydrate and fat (solid red arrow), the strong protein appetite ensures that absolute protein intake remains constant (vertical black line). Consequently, fat and carbohydrate intake increases (dashed red arrow) as does total energy intake (dotted red arrow) as a passive consequence of strong protein regulation

In a balanced food environment these macronutrient-specific appetites can all achieve their target intakes. If balanced diets are unavailable, the nutrient-specific appetites come into conflict, because in such circumstances (by definition) all regulated nutrients cannot simultaneously be ingested at their respective target levels. The outcome of this conflict will be determined by the relative strength of different appetites, with the stronger appetites more closely reaching their target intakes than weaker appetites. Studies using the ‘nutritional geometry framework’ [93] have shown that in humans and some other primates, protein is regulated more strongly than carbohydrates and fats [94, 95], and thus absolute protein intake remains relatively constant while fat and carbohydrate intake vary with the density of protein in the diet [92]. Accordingly, dilution of dietary protein by carbohydrate and fat results in the over-consumption of these nutrients, a scenario known as the ‘protein leverage’ of energy intake (Fig. 4) [92, 96].

Other biological mechanisms are also important. First, many foods and beverages incorporate psychoactive substances designed to tap into neurological ‘reward’ circuits that evolved in the context of much lower levels of stimulation [97, 98]. Similarly, foods rich in both carbohydrate and fat [99] and sedentary behavior can also affect appetite regulation and promote over-consumption [100]. As we show below, this means that altering the composition of foods provides opportunities to influence human agency through the mediating pathway of appetite [1, 101, 102].

**Fig. 5:**
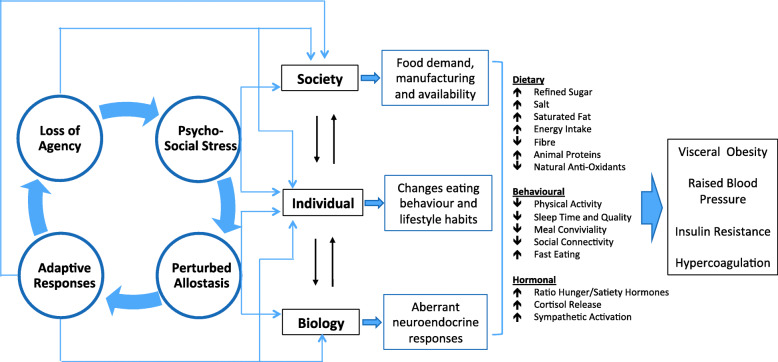
Impact of psychosocial stress on appetite, metabolism and the food system. Stressed individuals experience increased appetite, and consume high-energy palatable foods to dampen the stress response, under the influence of complex metabolic pathways involving the hormone cortisol and other signalling molecules. Within the body, these metabolic responses are associated with poorer cardio-metabolic profile, including insulin resistance, elevated blood pressure and greater susceptibility to blood clots (hypercoagulation). Insulin resistance and sustained increases in appetite also lead to excess weight gain, leading to chronically increased food intake. However, there are also many broader changes in behaviour, including perturbed sleep patterns and lower levels of physical activity, as well as faster eating behaviour and reduced sociality around meals. The interaction between stress and appetite generates an overall increased demand for high-energy palatable products, which drives greater supply, thus increasing the availability of unhealthy foods

However, this scenario is not restricted to the composition of food itself, and is also relevant to broader factors that influence human behavior. We focus here on psychosocial stress, which can impact both eating behavior and metabolic processing of the diet (Fig. 5**).** Experimental studies of rodents and humans demonstrate that consuming a high fat diet dampens the stress response, though at a cost of elevated NCD risk markers [103, 104]. These associations are attributed more strongly to the impact of the hormone cortisol on reward pathways and appetite centers in the brain, but there are many other components of signaling, including insulin, leptin, neuropeptide Y (NPY), endocannabinoids, gastrointestinal hormones and alterations of the microbiota [105, 106]. Several cohort studies have reported that the level of perceived stress is associated prospectively with BMI increase [107–109].

Chronic activation of the stress response is therefore interacting in many settings with the plentiful availability of high-calorie foods, thereby contributing to the rise in obesity [110, 111]. Importantly, stress is not experienced equally, and differential exposure to stress is a key factor mediating the relationship of societal inequities and inequalities with malnutrition. The role of stress biology is crucial for understanding both the etiology and the health impacts of the DBM, for through this medium the ‘competition of agency’ simultaneously structures the environments in which we eat, while also affecting how the body processes foods.

This nutritional ecology approach therefore frames nutrition as the interaction of biological mechanisms with the food environment, which sets the boundary conditions within which appetite systems operate [91]. This gives new insight into how societal factors drive malnutrition. For example, while overt dietary insufficiency drives weight loss, the ‘protein leverage’ hypothesis highlights how the aggressive marketing of highly processed foods can dilute protein intake, contributing to obesity [112]. Similarly, our appetite and metabolism respond to psychosocial stress, and to the social conditions in which we eat.

### Individual agency

When it comes to behavior, individuals do not explicitly maximize nutritional health, and instead pursue proximate goals such as satisfying hunger, obtaining affordable and palatable foods that are convenient to prepare, and conducting desirable activities [113–115]. Their nutritional status is shaped both by their ability to pursue these goals, and by the environments to which they are exposed and which therefore impact their agency. Individuals may employ substantial creativity, to try to balance their competing goals [116]. Poverty exacerbates such trade-offs and drives more severe deficits in health, by forcing agency to be targeted at satisfying basic economic needs.

The fundamental association of poverty with food insecurity, food shortages and poor quality diets has been recognized for millennia, indicating reduced agency to access a healthy diet. However, beyond dietary intake itself, the constraints on agency driven by poverty and low education [117, 118] also impact other stresses, such as exposure to pathogens and pollutants that impair growth and biological functions.

Structural drivers of malnutrition, such as poverty and inadequate education, inhibit both the agency and the means to improve household food insecurity and malnutrition. In turn, inadequate education (< 10 years) and in particular illiteracy, constrain women’s health, agency and opportunities to obtain better paid work which would enable the purchase of (typically costlier) healthier and diverse foods, and also to break out of poverty [119, 120]. Working long hours in the informal economy, or returning to school/vocational training (for younger mothers) also means that women have limited contact with infants, limiting the opportunity to breastfeed [121, 122]. Collectively, these structural factors not only maintain food insecurity, but also increase maternal stress and mental ill health and undermine their ability to fulfill their roles as mothers [123]. Left unaddressed, this cycle of disadvantage is likely to repeat across generations, whereby chronic malnutrition mediates the role of poverty in undermining physical health, cognitive development and academic ability [122, 124]. In the most severe conditions, individuals may assert agency against such constraints through collective action. For example, increases in food prices that threaten food security often provoke riots, especially among urban groups and when society has broader discontent with the status quo [125, 126].

Moreover, in contemporary populations these challenges are not experienced equally, and there are several groups whose agency over access to or selection of food is particularly prone to constraint. In poorer settings, for example, gender inequity may amplify these effects in women, who are often ascribed the most labor-demanding subsistence farming tasks [127], whilst being constrained in accessing adequately nourishing foods [128]. As an illustration of this, a study in Nepal identified gender differences in the household allocation of food, with men disproportionately consuming foods rich in animal proteins and important nutrients compared to women [129]. Another study in the same setting linked early marriage with shorter women’s height, suggesting a detrimental impact of psychosocial stress on linear growth [130]. The less women can meet the nutritional costs of reproduction, the more they transfer any nutritional insufficiencies to their offspring [131]. Consistent with that hypothesis, societal gender inequality assessed at the national level has been associated with higher rates of low birth weight and child wasting and stunting [132]. However, this scenario also relates to overweight as well as underweight, with women in countries with higher levels of gender inequality also at elevated risk of obesity [133].

At the level of geography, rural food producers tend to show higher rates of undernutrition than urban populations [51]. Farmers often lack agency over access to land, the ability to purchase agricultural inputs, and to decide on which crops or animals are farmed [43]. The returns on their labor are destabilized by ecological stresses and market volatility in commodity prices, both of which may demonstrate seasonal spikes [134]. Rwanda’s experience highlights how broader land consolidation and agricultural policies constrain the agency of poor households to obtain diverse nutritional diets [135]. Under this scheme, the government provides agrarian land for poor households to grow fruits and vegetables, and generate livestock products to sell at local rural markets. However, there is no mechanism to then facilitate the purchase of similar high nutrient foods for these households. Although poor households allocate 39% of their total monthly expenditure to food, the high price and poor availability of nutritious foods mean that they buy more of the cheaper low nutrient foods (e.g. high in starch/carbohydrates). Paradoxically, therefore, the greater proportion of time spent by women in growing healthy foods for markets, the higher the prevalence of child undernutrition [135]. Again, to overcome such challenges, food producers often mobilise collectively: the best-known example is the Via Campesina movement, an international alliance of peasant and family farmer organisations built from the bottom up. Via Campesina promotes ‘food sovereignty’ by make local agriculture and trade work more effectively in its members interests, improving outcomes for both food producers and consumers [136].

However, while rising incomes may increase agency over dietary intake by reducing the risk of food insecurity, they also increase exposure to commercial influences, and the resulting dietary shifts may lead to excess weight gain. Overweight typically first emerges among wealthier groups during nutrition transitions, but subsequently shifts to poorer groups, as cheap highly-processed foods are the most obesogenic [49, 137]. This helps explain why, while urban populations may be less susceptible to undernutrition, they have also been more prone to overweight [138].

**Fig. 6:**
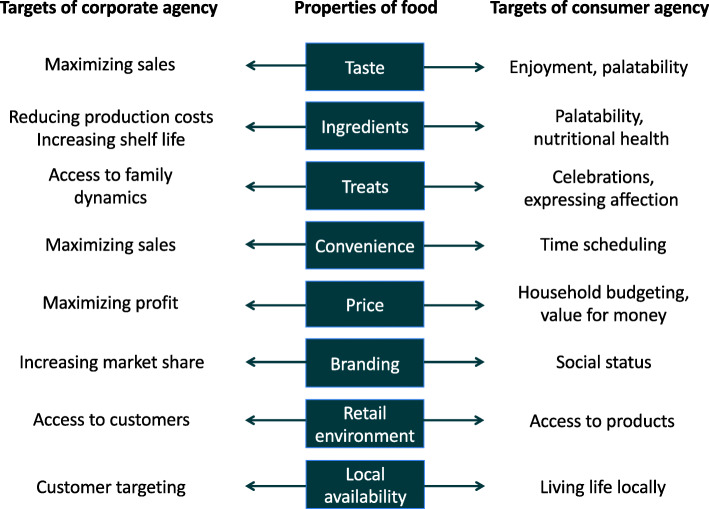
Differential targeting of food products by individual and corporate agency. Individuals express agency over their purchasing of food to satisfy a range of wants and needs, many of which are embedded in social or economic dynamics, and most of which do not relate directly to health. In contrast, corporate food producers maximise their agency over sales, and seek to manipulate a range of aspects of consumer behaviour to cut their production costs, increase their reach, and maximise their sales

Figure 6 lists a range of properties of foods that are actively targeted through the expression of agency of individual food consumers, but also by the agency of corporate food vendors. However, for each individual food property, what consumers and vendors seek to gain from expressing their agency is very different. For example, a food corporation pursues its goal of maximizing sales by manipulating the taste of a food product to maximize palatability. The consumer in contrast maximizes the goal of enjoyment, which may have both personal and social components, by purchasing foods considered tasty or adding to a harmonious meal [139, 140]. These contrasting approaches mans that although consumers may achieve a range of goals through their choice of foods, corporations are still able to influences these choices. The way in which they achieve this often steers the diet towards less healthy composition.

Individual agency is also influenced by local social norms, relating to cultural valuations of body image and foods. This means that alongside biological drives (appetite), there are also social drives to eat. In settings with limited food availability, larger body size and plumpness are typically markers of status or beauty, and processed foods are often seen as desirable symbols of modernity [141, 142]. The reverse pattern emerges in food-secure HICs, where slimness, leisure activities and the consumption of diverse fresh foods all signal status [143]. Changes in social norms therefore play a major role in dietary and behavioral change.

### Corporate agency

Commerce is embedded in every component of the human food chain, from agricultural production, shipping, marketing and retailing, to consuming. Corporate agency acts deliberately to reduce or distort individual agency in order to stabilize and increase commercial profits, but this comes directly at the expense of health, as recognized through the lens of the ‘commercial determinants of health’ [144].

Food processing adds economic value along the whole food chain. The most profitable foods are highly processed products that are easy to manufacture, ship and store, and that stimulate consumption by targeting both appetite and social status through branding [145]. Corporations compete over market share, and the most efficient firms expand in size at the expense of others. Consequently, market share is increasingly dominated by a handful of large TNCs [127], however economies of scale mean that individual food items remain relatively cheap to consumers. In LMICs, these pressures steer local food companies towards the same business models.

Commercialization of the food supply has reshaped the entire mode of eating [36], reducing emphasis on major meals and promoting inter-meal snacking, in particular on processed foods and beverages as well as alcohol. Retailers and food venues deliberately target unhealthy but profitable processed foods at large susceptible communities whose agency is most readily manipulated, as discussed below. Fast food outlets are often clustered in deprived neighborhoods [146, 147] and along school commuting routes [148, 149], while poorer urban populations may also be exposed to forms of ‘food desert’, lacking adequate access to healthier items [150].

Norms of social status are actively targeted to change dietary behavior [151], the primary targets being poorer groups in HICs and wealthier groups in LMICs [1]. In LMICs, advertising plays a key role in driving nutrition transition by portraying ‘new kinds of consumer’, an aspiration that can seemingly then be realized by consuming the relevant products [1]. However, corporate agency is achieved in part by creating ‘illusory agency’ for consumers, who are bombarded with substantial ‘choice’ over individual products, whilst simultaneously offered a range of foodstuffs that have in common high processing, low nutrient content and high profitability. These products have been specifically designed to manipulate agency over what, how much, and when food is consumed, following decades of research on palatability (hence manipulating appetite) and desirability (hence manipulating cultural preferences) [1, 101, 102]. In this way, corporations simultaneously target both biological and social drives relating to eating. It is precisely because people pursue a wide range of goals relating to food and eating that the food industry has targeted a wide range of opportunities to manipulate individual agency in the interest of corporate profit.

Beyond diet itself, commercial factors drive many other aspects of lifestyle related to obesity, including sedentary behavior through labor-saving devices, mechanized transport, and addictive digital activities. Similarly, the commercialization of agricultural inputs has radically shifted control from individual farmers to large agritech businesses, that now sell coordinated packages of seeds, fertilizers and pesticides [43]. Agritech corporations thereby restrict the range of crops grown by small-scale farmers to a fraction of the possible varieties [43], which perpetuates fundamental asymmetries between HIC and LMIC food systems. Cheap grains from subsidized industrialized farms in HICs are dumped in LMICs, undermining local food production, which in turn drives LMIC consumption of imported foods [152].

### Governments

Democratic governments should serve the interests of their voters by promoting health, through activities such as public health campaigns, taxing unhealthy foods, or regulating food composition, corporate advertising and nutrient-labeling [153]. However, their success in meeting these aims is limited by several factors. First, the financial resources available heavily favor corporations. In 2017, the leading 33 companies in the food, alcohol and tobacco sectors generated combined profits of USD 99 billion, an order of magnitude larger than the sum available globally for the prevention of undernutrition and NCDs [154]. Second, government agency is undermined by powerful corporate lobbying, and limited by the process of law, which gives many legal rights to corporations [155]. Third, corporations actively misinform and confuse consumers, thus negating education campaigns [156], and undermine public health research [157, 158].

Even in democracies, governments can themselves contribute to malnutrition through providing inadequate safety nets. Recently, austerity policies have been associated with a rapid increase in food banks in the UK [159], and with increased rates of child malnutrition in Spain [160]. However, while overt hunger has often provoked food riots [25], citizens rarely protest in favor of healthy foods [161]. Instead, food corporations have ‘manufactured consent’ for unhealthy products by making them ultra-palatable and cheap [1, 101]. This approach allows consumers to achieve agency over several goals – enjoying food, and obtaining value for money – though at a cost to their health. Consequently, the public often distrusts and resists public health campaigns, rejecting the assault on their agency to enjoy their diet and lifestyle. This is despite the fact that government regulation can cut NCDs substantially, as demonstrated by bans on transfats and smoking, and by salt reduction programs [162–164].

Non-democratic governments often use the medium of nutrition explicitly to control their populations. For example, autocratic regimes manipulate agricultural policy to maximize agricultural rents while minimizing the threat of unrest, and favor either urban populations, or landed elites and farmers, depending on where the threat is greatest [165]. Where such governments face active resistance, the resulting civil conflict may involve the deliberate imposition of food shortages to quell opposition [166]. Recent conflicts in Syria and Yemen highlight that sieges remain central to military strategy.

### Neoliberalism

Conceptual frameworks for public health nutrition often treat government as the ‘uppermost’ or ‘outermost’ level, and consider that nutritional health emerges from dynamics between governments, their citizens and commercial actors. Crucially, however, governments themselves are subject to a broader economic system, which since WWII has been increasingly restructured in support of a broader ‘neoliberal’ approach centered around competitive capitalism, consumerism, free trade, rapid trade liberalization, and minimum government regulation, all of which are considered by economists such as Friedman to be key prerequisites for political freedom [167]. Whether ‘neoliberalism’ is a paradigm, a political-economic project or an ideology [168–170] continues to be debated [171, 172], however as we show below, the specific policies supporting this approach have been widely implemented. The consequence has been to reshape the competition of agency amongst all the other actors.

In the 1970s, indebted LMICs undergoing economic crises were granted grants and loans by the World Bank and IMF with strict conditions coalescing around the central neoliberal principles. Since then, these practices have consolidated, and recent financial, food and fuel crises have resulted in the expansion of these ‘structural adjustment programs’ (SAPs), including to several European countries [173, 174]. SAPs are designed to enable countries to achieve macroeconomic stabilisation through controlling inflation, servicing debts to foreign creditors and stimulating economic growth. SAPs generally comprise of six types of policies: monetary, fiscal, exchange rate, foreign trade, wages and prices [175]. Since economic crises are largely blamed on state intervention, protectionism and price subsidies that distort market forces and undercut economic growth, SAPs specifically aim to dismantle these policies. In the continual effort to improve the efficiency of the public sector, contemporary SAPs also explicitly promote the privatisation of state assets, property and public services, and, in an effort to open domestic markets to foreign investment, they aim to lower TNC taxes, deregulate financial markets and expand trade liberalisation [86, 173, 176–178].

Seminal research by Pinstrup-Andersen [175] and more recent work by Babu and colleagues [179] have highlighted multiple pathways through which macroeconomic policies fundamentally restructure national economies, including food and agricultural policy, that then adversely impact household food security, income and the nutritional status of the poor in particular. Cornia and colleagues, writing for UNICEF, provided early evidence of how these adjustment policies worsened children’s nutritional status in Ghana, Jamaica, Peru, Philippines, Sri Lanka and Bolivia in the 1980’s [180]. Since then, evidence of the adverse effects of SAPs on nutrition and health has increased substantially [174], as we summarise below.

At the national level, SAPs have transformed domestic food systems and production by facilitating TNCs in diverting LMIC farmers from growing food to growing cash crops for export, thereby increasing household food insecurity [85, 181]. In order to drastically reduce inflation, repay debts and balance budgets in the short-term, countries have undergone stringent fiscal austerity. This has resulted in decreased government expenditure on welfare programs directly affecting nutritional status, such as food subsidies, and lowering or capping public sector wage bills, to the detriment of quality public health care and education provision, whilst simultaneously increasing the provision of these public services to either NGO or private providers [182–186]. Across LMICs, structural adjustment measures have been associated with falling real wages and increased household poverty, which, when coupled with rapidly increasing food prices, decrease purchasing power. As the overall consumption of food falls, households shift to purchasing cheaper, less nutritious foods, increasing risks of the DBM and infant and maternal mortality [180, 187–189]. Seasonable food shortages and imbalanced dietary intake also increase susceptibility to infectious disease, which often remains untreated because of decreased access to healthcare due to poverty [190]. Importantly, SAPs have increased women’s undernutrition and ill-health by simultaneously increasing their workloads in the reproductive and (often informal, lower paid) economic spheres; at the same time, rampant privatisation has decreased their access to agrarian land and support systems such as cooperatives, and the removal of State produce subsidies has undermined their ability to source adequate food [191–193].

More recent work has explored in more detail the different pathways through which, collectively, these policies and the institutions that implement them have systematically undermined governments’ capacity and agency to promote nutrition and health [194]. For example, an analysis of 141 LMICs from 1985 to 2014 finds that specific conditions on privatization, price deregulation and public sector employment have had a negative effect on the capacity of the state to effectively implement development policies that in turn could sustain economic growth [195] – the latter ironically being a key goal of SAPs [182–186, 195]. In contrast, Dollar and Svennson’s (2000) review of 182 World Bank adjustment loans suggested that the main reason for the 36% failure rate was due to the recipient country’s authoritarian political-economy. Hoey proposed that in Bolivia, efforts to decentralize and downsize government, and the rapid proliferation of NGOs delivering public provision, eroded state capacity to effectively deliver programs to reduce malnutrition [196]. In Fiji, trade and investment liberalization promoted by SAPs increased the power of multinational food and beverage companies to redefine the domestic food market. Initially, marketing regulations prohibiting the promotion of formula feeding led to companies retracting these products, which ironically led to consumer protest over reduced ‘choice’ [197]. The regulations were not only rescinded, but in 2016–17, the import of infant food became ‘duty free’ [197]. Through several different pathways, therefore, SAPs systematically undermine the capacity of states to promote the nutritional health of their citizens.

Within the neoliberal system, market and trade liberalization has been central to the LMIC ‘nutrition transition’ that has played a fundamental role in the emergence of the DBM [198]. Broadly, the WTO and other instruments such as Trade and Investment Agreements have reshaped the whole spectrum of food systems, impacting food production, manufacturing, distribution and marketing [199, 200]. ‘Power hungry,’ a report by the NGO ActionAid International, documents several ways in which global food companies control domestic agrifood markets, from seed to supermarket, and remain unaccountable for their negative impacts on farmers’ livelihoods, the human right to food, and the environment. Their activities include raising the price of agricultural inputs; engaging in unfair buying practices, including price-fixing cartels; lowering prices for farmers’ goods, which decreases producer income whilst maintaining high retail prices, thereby increasing corporate revenue; and marginalizing poor farmers and rural workers from the supply chain and access to justice [127]. Food prices, supply and availability directly affect child nutritional status by decreasing the revenue of agricultural producers, thereby affecting household income and changing the types and amount of food purchased and consumed. In contrast, government assistance to tradable agriculture through reduced taxation was found to improve child nutritional status across 22 LMICS [201].

More specifically, trade liberalization has contributed directly to the escalating obesity pandemic, in part through expanding imports of highly-processed foods [198, 202]. For example, WTO arrangements have been associated with substantially higher intakes of sweetened beverage across LMICs [10, 203, 204], while across African countries, the percentage of food that is imported correlates with adult obesity prevalence [205]. As with SAPs, the agency of LMIC governments to promote nutritional health and public health oversight is actively challenged by the legal rights accorded to TNCs to protect their investment in trade agreements [206–208]. For example, Thailand abandoned a proposal to initiate a healthy food labeling system [209], whilst Vietnam dropped a tax on sweetened beverages for fear that corporations could sue the government for potential loss of earnings [203]. Even when governments are able to defeat such lawsuits, they may be left with crippling legal costs [210].

**Fig. 7:**
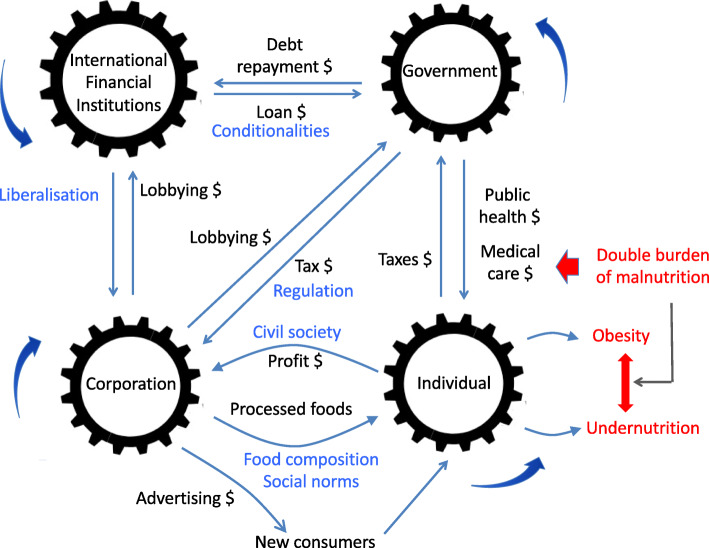
The political and commercial determinants of nutritional health. The nutritional status of individuals is strongly shaped by asymmetric power dynamics and financial flows among a set of actors, including corporations, governments and supranational organisation. In contemporary food systems, these dynamics drive the double burden of malnutrition. Black text – financial flows; blue text – power relations; red text – markers of ill-health

Based on the evidence presented above, we conclude that there is a hegemonic neoliberal economic orthodoxy, which has been implemented in a concerted manner to restructure the world economy, most notably through technical policy mechanisms designed under SAPs of the IFIs, or through the WTO and trade agreements, which have transformed welfare states into competitive states [211, 212]. Irrespective of the country, the neoliberal approach has exacerbated inequity in nutrition, health and educational outcomes, and has reduced the capacity of states to respond by fulfilling these basic rights more broadly. This overall reduction in human capital has on the one hand failed to achieve the central neoliberal goals of debt reduction and economic growth, but on the other hand succeeded in transferring wealth and capital to wealthy corporations and nations. Collectively, these examples show that neoliberalism functions at multiple levels and in multiple forms, involving global institutions, nation-states, and corporations [213]. We highlight that it is the way in which neoliberalism has reshaped the ‘competition of agency’ that accounts for its fundamental role in the emergence of the DBM (Fig. 7).

Perhaps most concerning is how the entry of cheaper, less nutritious foods has become a space of activism, with consumers paradoxically demanding their right to choose these less healthy products. These actions are in large part driven by lower income and limited control over food environments [197], and reflect the way that individual agency has been undermined by the manipulation of both purchasing power and appetite/palatability. It is precisely the way in which neoliberalism is increasingly embedded at every level of society and economy which makes it both difficult to address, but all the more important to challenge in order to achieve equity in nutritional health. This progressive erosion of social citizenship has provided the impetus for collective mobilization against neoliberalism over the past four decades [214].

### The competition of agency

**Fig. 8:**
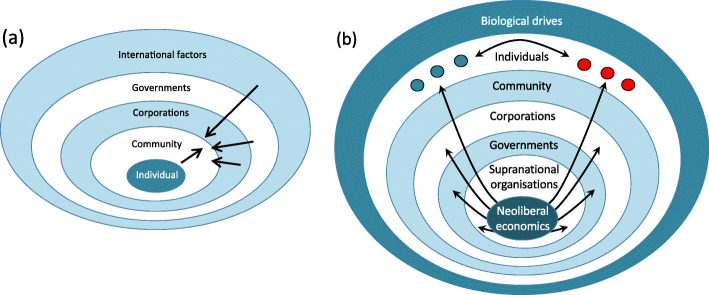
Contrasting socio-ecological models of nutrition and agency. (**a**) The individual-centric view emphasises the individual, whose behavioural agency drives interactions with the social community, corporations and government activities. (**b**) The system-centric view emphasises the food environment as a system shaped by the logic of market economics. The overall system shapes government and corporate activities, and generates structural associations between different socio-economic groups (shown by red or green filled circles), whose biological drives are exposed to contrasting nutritional experience through the life-course

This review of agency as a ‘competition’ between multiple actors highlights how individuals are exposed to both commercial and political determinants of nutritional health [215]. Nutritionists have long gained insight from socio-ecological models that provide an individual-centric view [216], focusing primarily on behavioral interactions between individuals, corporations and governments. Following the logic of Swinburn and colleagues [5], we can gain a very different perspective by flipping the model, putting the politico-economic system center-stage (Fig. 8). Our alternative model contains more layers, and enables us to see malnutrition as the outcome of interactions between human metabolism and many forms of power dynamics deeply embedded in the global food system. The model also helps understand the many inequalities that produce contrasting nutritional outcomes across different social groups. The main value of our approach lies in the fact that, as we have highlighted above, there is now substantial evidence for interactions between all the different layers. We show below, moreover, how a novel ongoing stress is demonstrating in real time the sensitivity of human malnutrition to rapid changes in these dynamic relationships.

By presenting this competition of agency, we also highlight the inadequacy of existing models of agency at the individual level. Growing understanding of how food composition impacts satiety, how eating patterns respond to social factors, and how psychosocial stress impacts both appetite and metabolism, forces us to question concepts of agency that define it in terms of other individual-level factors such as liberty and autonomy. Similar challenges relate to other aspects of behavior, such as physical activity and sedentary behavior. What are often portrayed as individual ‘choices’ are in reality better considered acts of behavior where choice is suppressed or manipulated. As we have argued previously, it is precisely because nutrition, metabolism and cognitive function represent an interface between corporate agency and individual agency that our understanding of individual agency in the context of nutrition is problematic [1]. The same scenario applies to the interactions of individuals with other actors, such as governments and IFIs. Without better understanding of this issue, we will struggle to promote individual level agency and empower public health programs, or curtail the agency of other actors central to the DBM and its associated effects on ill-health.

## Lessons from the COVID-19 pandemic

The utility of our framework for understanding the future burden of malnutrition is supported by emerging evidence of the effects of the COVID-19 pandemic. The dynamic interactions of food and economic systems with individual agency and biology are giving rise in every global region to shifts in food insecurity, diets and eating behavior, however the effects are variable and depend on the local context. The pandemic highlights the inability of the global food system to protect populations either from hunger, or from diet-influenced NCDs, whilst also showing that malnutrition increases susceptibility to this disease [217].

Even in high-income countries, those who have experienced job loss or income insecurity have undergone increased exposure to food insecurity. Use of food banks has increased [217, 218], while the practice of food hoarding temporarily reduced the availability of food for low-income families who cannot afford to buy in bulk [219]. Policies of isolation and ‘lockdown’ have in many cases increased the consumption of unhealthy foods, as well as unhealthy commodities such as alcohol and cigarettes, and some groups have experienced excess weight gain. Levels of physical activity have often declined, in association with limited access to recreational facilities. However, early data also indicate substantial heterogeneity in these responses, as some groups have experienced confinement without substantial change in economic circumstances.

Studies in European settings have shown that those already overweight tended to gain further weight, with psychosocial stress stimulating increased consumption of energy-dense ‘comfort foods’, whereas those already underweight have tended to lose weight [220–222]. In Italy and Spain, however, lockdown policies increased the prevalence of home cooking, promoting overall adherence to the healthy Mediterranean diet, while the consumption of savory snacks, processed meat, and carbonated/sugary drinks decreased [220, 223]. Some, especially those already exercising, tended to adopt new exercise regimes, while those already sedentary tended not to change their status [224]. Similarly, some have quit smoking, whereas others have consumed more alcohol and increased smoking [222, 224].

An international global survey in 7 languages, involving 1047 individuals primarily from Africa, Asia and Europe, found that home confinement reduced physical activity at every level of intensity, and led overall to less healthy patterns of food consumption and meals [225]. Another study of adolescents from Spain, Italy, Brazil, Colombia, and Chile found that confinement was associated on the one hand with more time for cooking and greater consumption of legumes, fruit, and vegetables, but also with higher sweet food consumption which was attributed to boredom and stress [226].

In lower income countries, there is risk of major disruption to many sectors fundamental to nutrition, including food supply chains and markets, income, social protection, health care services for women and children, and access to clean water and sanitation [227, 228]. The effect of simultaneous loss of income with increases in food prices may be particularly detrimental to those working in urban informal economies. As yet, there are few published data on the numbers affected or the magnitude of the effects, but according to preliminary projections, UNICEF has predicted that ‘the COVID-19 pandemic may add an additional 83 to 132 million people to the ranks of the undernourished in 2020’ [229]. These stresses may generate particularly severe impacts on those already susceptible to food insecurity, for example migrant workers or those living with HIV [230].

The efforts of food corporations to manipulate individual agency in their own interest during the pandemic has also been observed. In India, for example, there is evidence of an infant formula manufacturer using social media to recommend the separation of mothers with COVID-19 from their infants for 72 h and to stop breast-feeding, despite this contradicting both Indian law and medical advice [231]. Corporations have used the pandemic and the rapid changes in eating patterns as a marketing opportunity [232], and among adolescents, the intake of highly processed foods was found to have increased in each of five countries surveyed in Europe and South America [233].

Finally, it should be noted that COVID-19 also has a bi-directional association with nutritional status. Obesity has already emerged as a strong risk factor for COVID severity and mortality [234–236], while impaired immunity associated with nutritional deficiencies may also increase the risk of infection and poor prognosis [237].

Overall, the impact of COVID-19 on food systems combined with lockdowns have greatly reduced individual agency for many, but the same factors have sometimes also reduced the agency of corporations in the short-term to vend unhealthy products or fast food. Of note, none of these events or policies deliberately targeted nutritional health, and yet changes in the prevalence of both undernutrition and overweight are likely to emerge as responses.

## Future outlook

Over recent centuries the world has witnessed unprecedented economic growth, scientific progress and technological development. However, the benefits have not been shared equally, while the costs are manifesting as climate breakdown, environmental degradation and persisting malnutrition. The global food system developed in part through the function of maintaining societal inequalities, and the contemporary DBM reflects these dynamics. If we consider the global food system as a ‘game’ that requires solving, then there is a rapidly diminishing landing space for a solution that is simultaneously equitable and healthy for people and planet.

Sen’s insight that famines represent failures of society rather than food production [7] is highly relevant to the DBM. However, while progress has been made in increasing access to food, compromised agency at the level of the individual as well as the state continues to be crucial in understanding malnutrition, whatever its manifestation. How to maximize nutritional health therefore remains a major challenge.

### Technical opportunities

Technical advances concern changes in the composition of food, and therefore either ignore individual agency, or seek actively to bypass it. This can potentially have advantages in certain situations, for example altering the composition of foods used to treat undernutrition may bypass poor appetite and accelerate nutritional recovery. The same approach could in theory also be used in reverse to combat obesity, however to date the food industry has shown very limited engagement with this opportunity, highlighting how their own agency and interests contribute to this health problem.

Regarding childhood undernutrition, the development of ready-to-use therapeutic foods (RUTFs) has played a key role in reducing mortality rates whilst also promoting weight recovery [238]. Containing no water, RUTFs prevent microbial growth and can be eaten directly from the sachet, allowing children without complications to be treated in their homes. However, the gut microbiota of severely undernourished children is immature, a scenario only transiently improved by RUTF treatment. Future work might identify complementary foods that could help ‘repair’ the immature microbiota and promote healthier growth [239]. Producing such RUTFs locally/regionally, and basing them on locally available microbiota-directed ingredients, may make them more efficient, culturally acceptable and sustainable. For less severely undernourished children, smaller amounts of RUTFs might also support a healthy gut microbiota, again benefitting growth. Recently developed food supplements for undernourished children primarily promote the accretion of lean mass rather than fat [240], reducing fears that treating short and underweight children might increase the risks of obesity and NCDs [241].

Regarding obesity, there is growing evidence that altering diet composition could potentially inhibit excess weight gain through effects on appetite. For example, the framework of nutritional geometry suggests that increasing dietary protein and fiber content could reduce passive energy consumption. To date, however, lifestyle interventions targeting individuals have generally had limited impact, largely because they are too easily countered by corporate agency. The food industry manufactures numerous ‘diet products’, but these do not lead to sustained changes in appetite, and are more effective in changing food purchase habits than in reducing body weight. Pharmacological solutions to obesity have also proven challenging, as there is no central metabolic pathway for drugs to target, though combination therapies are currently attracting interest [242]. The most effective therapies for significant prolonged weight loss are surgical operations [243], which cause unpleasant side effects and overwhelm health services.

In each case, technical development has primarily been directed to the treatment of malnutrition and its co-morbidities after the condition has developed. Progress in prevention remains relatively limited, the best example being micronutrient fortification programs that can reduce micronutrient deficiencies [244]. A criticism of this approach is that it provides opportunities to extract profit from the loss of individual agency over health, but from another perspective it could also be seen to improve agency over factors that promote nutritional deficiencies, and may be a very effective component of public health programs.

### Societal opportunities

A rebalancing of agency represents the most powerful solution to the DBM. This requires that we elucidate in more detail the many components of power dynamics in the human food system, and clarify the conflicts of interest between the actors, in order to identify novel targets for intervention. As we have shown above, power dynamics at many levels play a central role in the rising prevalence and unequal distribution of all forms of malnutrition.

Hindering our ability to alter these power dynamics, however, are many ways in which the status quo is perpetuated. For example, within any country or community, gender inequality is underpinned by societal norms that must be actively challenged. Similarly, corporations have not only acquired legal protections similar to those of individual citizens, but also additional rights that render them largely autonomous from public control [245]. In the same vein, the larger economic system, including influential IFIs, is not subject to the kinds of scrutiny or regulatory mechanisms that pertain to governments, while TNCs are likewise unconstrained by international laws that protect vulnerable populations [215].

The level of IFIs is rarely considered as a potential target for intervention in nutrition policy, yet as we have reviewed above, evidence for its impact is now strong. Citing influential work by Farmer [246–248], Pfeiffer and Chapman argued that if ‘... social and economic rights are human rights, [then] the role of a robust public sector and government emerges as vital; not sufficient, but necessary to guarantee the right to survive. Viewed in this light, structural adjustment’s systematic dismantling of public services for health, education, agriculture, water, and safety nets is rightly seen as a war on the poor; its violence measured in increased morbidity, excess mortality, … and the harder-to quantify destruction of community … ’ [174]. Our argument is that malnutrition is a particularly sensitive lens through which such damage can be assessed.

Reflecting these power inequalities, there are increasing calls to address malnutrition by strengthening human rights [249]. In 1996, a UN charter outlawed the control of food distribution for political ends, yet the imposition of food restriction for political and military ends has continued in each continent in the early twenty-first century [166]. More generally, global food policies are deeply embedded in multiple economic activities that serve political ends. Moreover, although the ‘right to food’ is widely recognized, providing ‘adequate’ food represents a relative standard, open to debate and distortion [250]. The Convention of the Rights of the Child commits signatories to regulate unhealthy food advertising and promotion, but this may be undermined by cultural rights that protect ‘values, beliefs, convictions … and ways of life’, all of which may be targeted by corporate efforts to shape social norms [251]. The ‘capability approach’ of Sen and Nussbaum, already applied in other disciplines such as education and gender inequity, could also guide us in combatting the DBM [252] if we can identify effective strategies for transforming inequitable social power hierarchies [253]. The neoliberal system has narrowed our definition of human development, and has resulted in maximizing short-term economic growth at the cost of human and planetary health. Economic growth should not be considered an end in itself, rather as only a means towards increased wellbeing of populations and human development. This ‘means versus ends’ confusion has contributed to persisting inequalities, and the capability approach provides us with a legal framework that can guide us in combatting the DBM.

More broadly, existing rights frameworks are ill-equipped to deal with TNCs and IFIs, because government obligations are limited to their own territory. A new legal framework is needed on the extraterritorial application of human rights and specifically the obligations of supranational organizations.

However, we should also remember that power dynamics represent only one component of the human food system, and that other ecological, agricultural, biological, socio-cultural and economic factors are also relevant. Human nutrition represents a nexus of complex systems, in which there is no natural balance between the various hierarchical levels or actors, and where individual components may be connected by non-linear associations and by complex feedback loops. Systems theory may offer new ways to understand how changes in one aspect of the system are likely to affect others, and this concerns both power hierarchies and other ecological factors. For example, the association of dietary protein with appetite is expected to be sensitive to rising CO_2_ levels, which have been found to dilute the protein, fiber and micronutrient content of vegetable crops with starches [254, 255]. Better understanding of these inter-relations, including how the ‘competition of agency’ interacts with broader ecological factors, will facilitate interventions while avoiding unintended consequences, and may help identify how power dynamics may be targeted in order to achieve the greatest benefits.

## Conclusion

Malnutrition, social inequity and inequality, and climate breakdown each manifests as an existential crisis for our species. Crucially, each is determined by a broad common set of political and commercial interests that override individual agency. However, to date scientists have struggled to develop broader conceptual models that are capable of expressing and exploring these interactions. This has hindered researchers and policymakers from gaining appropriate evidence on the deeper structural causes of these problems, and hence from developing appropriate policy responses. Existing physiological models of the causes and manifestation of malnutrition have remained largely disconnected from socio-ecological or economic models of food systems. In this paper, we aimed to address this lacuna, by setting out a new integrative conceptual framework that describes in detail how societal factors impact and interact with human physiology, thereby determining variability in nutritional status and the risk of all forms of malnutrition. For example, we are able to frame how international economic policies and corporate practices, mediated by food composition (eg protein content) and social factors (eg exposure to stress), shape dietary intake and nutritional status. This sheds new light on the international structural drivers of the global obesity epidemic and persisting under-nutrition.

This approach allows us to highlight a competition of agency between a range of different actors as the essential target of efforts to prevent malnutrition in the future. Given the foundational role of food systems in all human societies and ecosystems, the rebalancing of agency that we describe above must be central to tackling not only malnutrition, but also social inequality and climate breakdown. In the words of the former UN Secretary General, Ban Ki-moon, ‘Nutrition is both a maker and a marker of development. Improved nutrition is the platform for progress in health, education, empowerment of women and the reduction of poverty and inequality, and can lay the foundation for peaceful, secure and stable societies’ [256].

## Data Availability

Not applicable.

## Abbreviations

BMI: Body mass index
DBM: Double burden of malnutrition
HIC: High-income country
IFI: International financial institution
LMIC: Low- and middle-income country
NCD: Non-communicable disease
NGO: Non governmental organization
NPY: Neuropeptide Y
RUTF: Ready-to-use therapeutic food
SAP: Structural adjustment programs’
TNC: Transnational corporations
WTO: World Trade Organization
